# Anti-heat stress lick block supplementation alleviated the detrimental effects of heat stress on dairy cows

**DOI:** 10.3389/fvets.2025.1562964

**Published:** 2025-03-07

**Authors:** Hongwei Duan, Jiyou Zhang, Na Li, Liuping Chen, Danhong Chen, Hang Yang, Qiuxia Dai, Junshi Shen, Shengyong Mao

**Affiliations:** ^1^Laboratory of Gastrointestinal Microbiology, National Center for International Research on Animal Gut Nutrition, Nanjing Agricultural University, Nanjing, China; ^2^Ruminant Nutrition and Feed Engineering Technology Research Center, College of Animal Science and Technology, Nanjing Agricultural University, Nanjing, China; ^3^China Salt Jintan Co., Ltd., Changzhou, China

**Keywords:** heat stress, dairy cow, lick block, lactation performance, heat stress index, rumen fermentation

## Abstract

**Introduction:**

Heat stress poses a significant challenge to the development of dairy industry, affecting cows’ well-being and overall productivity, leading to substantial economic losses. In this study, the impact of a specifically formulated anti-heat stress lick block supplement on milk production, milk quality, feed intake, rectal temperature, respiratory rate, and rumen fermentation in cows exposed to heat-stress was evaluated.

**Methods:**

Twenty-four healthy Holstein lactating dairy cows were divided into two blocks based on milk yield (low and high), Parity (2–3 parity), and lactation days (114 ± 8 d). The cows in each block were randomly assigned to either a control group without lick block supplementation or a treatment group receiving lick block. The trial lasted for 6 weeks, including a 2-week adaptation phase followed by 4 weeks of feeding treatment.

**Results:**

Heat stress levels varied from severe (THI > 88) to moderate heat stress (THI > 80) in the first 2 weeks, gradually decreasing to mild heat stress (THI > 72) in the following weeks. With the decrease in heat stress, dry matter intake (DMI) and milk production increased (Week: *p* < 0.05), the rectal temperature and respiratory rate of cows decreased (Week: *p* < 0.05). Lick block supplementation tended to increase DMI (*p* = 0.09), and improved milk yield (*p* < 0.05) without affecting (*p* > 0.05) milk composition, leading to increased milk yields of fat, protein, and lactose (*p* < 0.05). Although the overall rectal temperature of cows in the lick block group did not differ from the control group (*p* > 0.05), the respiratory rate of cows in the lick block group significantly decreased (*p* < 0.05) in the second and third weeks. Supplementation with the lick block increased (*p* < 0.05) rumen pH and decreased (*p* < 0.05) NH_3_-N and propionate concentrations in dairy cows, and tended to lower the acetate-to-propionate ratio (*p* = 0.07), total VFA concentration (*p* = 0.07), and butyrate concentration (*p* = 0.09).

**Conclusion:**

Supplementation of anti-heat stress lick block alleviated the detrimental effects of heat stress on dairy cows within a certain range of temperature and humidity.

## 1 Introduction

Holstein cows exhibit robust adaptability to cold but show poor tolerance to heat. High temperatures and humidity can disrupt the cows’ physiological equilibrium, leading to heat stress (1, 2). Previous studies have demonstrated that temperatures exceeding 25°C can easily induce heat stress in dairy cows, resulting in metabolic disorders, endocrine dysfunction, and negative impacts on the production performance, and health of dairy cows (3–5). In recent years, with the continuous growth of milk production, the heat generated by cows themselves has also been increasing. Additionally, the intensification of the global greenhouse effect has further increased the susceptibility of cows to heat stress. The thermal humidity index (THI) threshold, previously utilized to assess heat stress in cows, has been reduced from 72 to 68 (6). Gunn et al. predicted that milk yield losses across the United States will accelerate, with an average rate of 174 ± 7 kg/head/decade, due to the challenge of heat stress (7). Ranjitkar et al. also predicted that milk yield in China may decrease by 6.5 kg/head/day in 2050, with losses increasing to 7.2 kg/head/day in 2070 (8). At this rate, it must cause huge economic losses to the dairy industry and seriously hinder its healthy and sustainable development. Therefore, finding effective means to alleviate heat stress in dairy cows and reduce its effects on production performance and health is very important.

The current dairy farms primarily employ physical solutions like fans, sprinklers, and wet curtains to cool the cowshed, which were widely adopted and cost-effective practices in ranches (9). Additionally, some nutritional strategies are leveraged to further mitigate heat stress in cows including improving dietary energy levels (10), incorporating feed additives (11), and adding traditional herbal supplements (12). Several recent studies also point to the potential of various mineral elements, micronutrients, and vitamins in alleviating heat stress. For example, increasing the level of potassium (K) and sodium (Na) in the diet can enhance dry matter intake and milk yield of dairy cows exposed to heat stress, alleviating the detrimental effects of heat stress (13). Chromium (Cr) has been demonstrated to enhance cows’ production performance, immune function, glucose metabolism, and antioxidant capacity. Supplementation with an appropriate dose of Cr could alleviate the adverse effects of heat stress and enhance production performance (14). Vitamin C serves as a stress protectant for livestock (15), while vitamin E acts as a biological antioxidant and free radical scavenger, safeguarding cells and lipid-rich membranes from oxidative damage induced by heat stress (16). Furthermore, vital trace elements such as zinc (Zn), copper (Cu), manganese (Mn), and selenium (Se) have been shown to have the potential to alleviate heat stress (17). The mineral and trace elements in the diet can usually meet the production and health needs of cows. However, heat stress can lead to a decrease in feed intake and result in picky eating behavior. Therefore, it is necessary to supplement additionally to alleviate the effects of heat stress.

Lick block is a feed product made by combining salt, minerals, vitamins, and other nutrients in a specific proportion. Livestock can freely lick according to their health condition and physiological needs to supplement specific nutrients for livestock (18). At present, in countries with developed animal husbandry such as the United States, the Netherlands, and Australia, feeding lick blocks have become a necessary supplementary feeding technique for grazing and farming livestock. The Food and Agriculture Organization of the United Nations (FAO) has also promoted this low-cost and fast-acting technology to over 70 countries in Asia and Africa, achieving significant success (19). Supplementing lick block can promote saliva secretion in cows, facilitate digestion and rumination, alleviate rumen acidosis, maintain rumen acid–base balance, and be beneficial to the health of cattle and sheep (20). This experiment designed an anti-heat stress lick block targeting the needs of cows for minerals, trace elements, vitamins, and other nutrients under heat stress, and subsequently conducted an animal experiment to evaluate its anti-heat stress effect.

## 2 Materials and methods

### 2.1 Animals, diets, and experimental design

The Animal Care and Use Committee of Nanjing Agricultural University approved the experimental procedures used in this study (Protocol number: SYXK2017-0007).

Twenty-four healthy Holstein lactating dairy cows were divided into two blocks based on milk yield (low block: milk yield = 15.72 ± 1.46 kg; high block: milk yield = 20.65 ± 2.44 kg), Parity (2–3 parity), and lactation days (114 ± 8 d). The cows in each block were then randomly assigned to two groups: (1) CON group: not offered lick block; LB group: offered lick block. The anti-heat stress compound nutrition (HSCN) lick block used in the experiment was cooperatively designed by Nanjing Agricultural University and China Salt Jintan Co. Ltd., and the compositions are shown in Supplementary Table S1. The HSCN lick block primarily provides minerals and vitamins that contain NaCl, 894 g/kg; K, 15 g/kg; Co, 75 mg/kg; Cu, 500 mg/kg; I, 75 mg/kg; Fe, 2200 mg/kg; Mn, 2000 mg/kg; Se, 25 mg/kg; Zn, 4000 mg/kg; Cr, 15 mg/kg; VC, 3000 mg/kg; VA, 200000 IU/kg; VD, 80000 IU/kg; VE, 2000 IU/kg. All dairy cows were housed in indoor pens measuring 1.2 m × 2.2 m, featuring woodchip bedding, and had ad libitum access to fresh, clean water. Dairy cows were fed three times daily (0630; 1430; 2030) with the total mixed ratio (TMR) formulated by the NASEM standards (21), and the composition and nutrient content of the TMR are shown in Table 1. Feeding trials were conducted for 6 weeks, consisting of 2 weeks for adaptation followed by 4 weeks of feeding treatment.

**Table 1:** Ingredient and chemical composition of the basal diet.

Item	The basal diet
Ingredient (% of DM)	
Corn silage	28.00
Chinese wild rye	10.50
Alfalfa hay	13.00
Corn grain	20.00
Soybean meal	10.00
DDGS	6.00
Wheat bran	3.00
Caramel meal	6.00
Premix^1^	2.50
Sodium bicarbonate	0.80
*Saccharomyces cerevisiae*	0.15
Mould inhibitor	0.05
Nutrient composition (% of DM)	
DM	49.95
CP	15.46
RDP	10.26
RUP	5.20
NDF	32.26
ADF	18.11
EE	3.41
Ash	9.75
NE_L,_ Mcal/kg	1.61

^1^Formulated to provide (per kilogram of premix): NaCl, 160 g; Ca, 175 g, P, 30 g; Mg, 30 g; VA, 340000 IU; VE,65000 IU; VE, 1500 IU; Cu, 600 mg; Co, 12 mg; I, 40 mg; Se, 20 mg; Zn, 2,760 mg; Mn, 1, 600 mg.

### 2.2 Sampling and measurement

#### 2.2.1 Temperature and humidity index

The temperature and relative humidity in the barn were recorded at 0700 h, 1,400 h, and 1900 h daily throughout the experimental period using an automatic temperature and humidity recorder (TH20R; Shenzhen Huahanwei Technology Co., Ltd., Shenzhen, China). The THI was calculated according to the Specification for the Technical Assessment of Heat Stress in Dairy Cows (Ministry of Agriculture of the People’s Republic of China, 2013). THI = 0. 81*T + (0. 99*T-14.3) * RH + 46.3, where T = ambient temperature in °C and RH = relative humidity expressed as a proportion, i.e., 75% humidity is expressed as 0.75.

#### 2.2.2 Respiratory rate and rectal temperature

The respiratory rate (RR) and rectal temperature (RT) of the cows were measured at 0700 h, 1,400 h, and 1900 h twice a week. RR was measured according to the movement of the abdomen and thorax of the cows for 1 min and repeated twice. RT was measured using an animal thermometer (Dong-e–e-jiao Ahua Medical Instrument Co., Ltd., Jinan, Shandong, China).

#### 2.2.3 Feed intake and diet composition

Feed intake was measured and feed samples were collected twice a week throughout the experimental period. The samples were mixed, dried at 60°C for 48 h, and then crushed for determination of dry matter (DM), crude ash (Ash) and crude protein (CP), crude fat (CF) content in the samples according to the methods of AOAC (22). Neutral detergent fiber (NDF) and acid detergent fiber (ADF) content were analyzed according to the method described by Van Soest et al. (23).

#### 2.2.4 Milk yield and composition

Milk yield and composition were measured on the last 2 days of each week. Milk yield was measured using a Tunisian flowmeter (JHF-G17, Sichuan Jinhaifeng Animal Husbandry Technology Co., Ltd., Sichuan, China) during the three daily milkings. The flowmeters were calibrated before use. The milk samples were preserved with 2-bromo-2-nitropropane-1,3 diol and stored at 4°C. Milk samples collected three times daily were mixed at a ratio of 4:3:3. The milk composition was determined using a near-infrared analyser (MilkoScanTM 7RM, Foss Electric, Denmark) following the standard procedure.

#### 2.2.5 Rumen fermentation parameters

At the end of the trial, rumen fluid was collected using an oral stomach tube according to the method described by Shen et al. (24). Once collected, rumen fluid pH was measured immediately using a pH meter (Ecoscan pH 5, Eutech Instruments, Singapore). After filtering with four layers of gauze, the rumen fluid was dispensed and stored at −20°C for subsequent VFA analysis. 1 mL of rumen fluid was thawed with running water and the NH_3_-N concentration was determined calorimetrically using ammonium chloride as standard (25). Volatile fatty acids (VFA) were determined using a gas chromatograph (Agilent 7980A, United States) using the method described by Shen et al. (26).

### 2.3 Statistical analyses

Data for DMI, milk yield, milk composition, RR, and RT were analyzed using the SAS Mixed procedure of SAS version 9.4 with week as repeated measures using compound symmetry covariance structure selected based on Akaike’s information criterion for optimal fit. The model included fixed effects of treatment, block, week, treatment × week interaction, treatment × block interaction, as well as random effect of dairy cow within treatment × block. Data for rumen fermentation parameters were analyzed using the SAS Mixed procedure of SAS version 9.4. The model included fixed effects of treatment, block, treatment × block interaction, and random effect of dairy cow within block × treatment. The freedom degrees were determined utilizing the Kenward-Roger option. *p <* 0.05 indicated significant differences, and 0.05 ≤ *p* ≤ 0.10 indicated a trend of variation.

## 3 Results

### 3.1 Environmental temperature and humidity index

**Figure 1:**
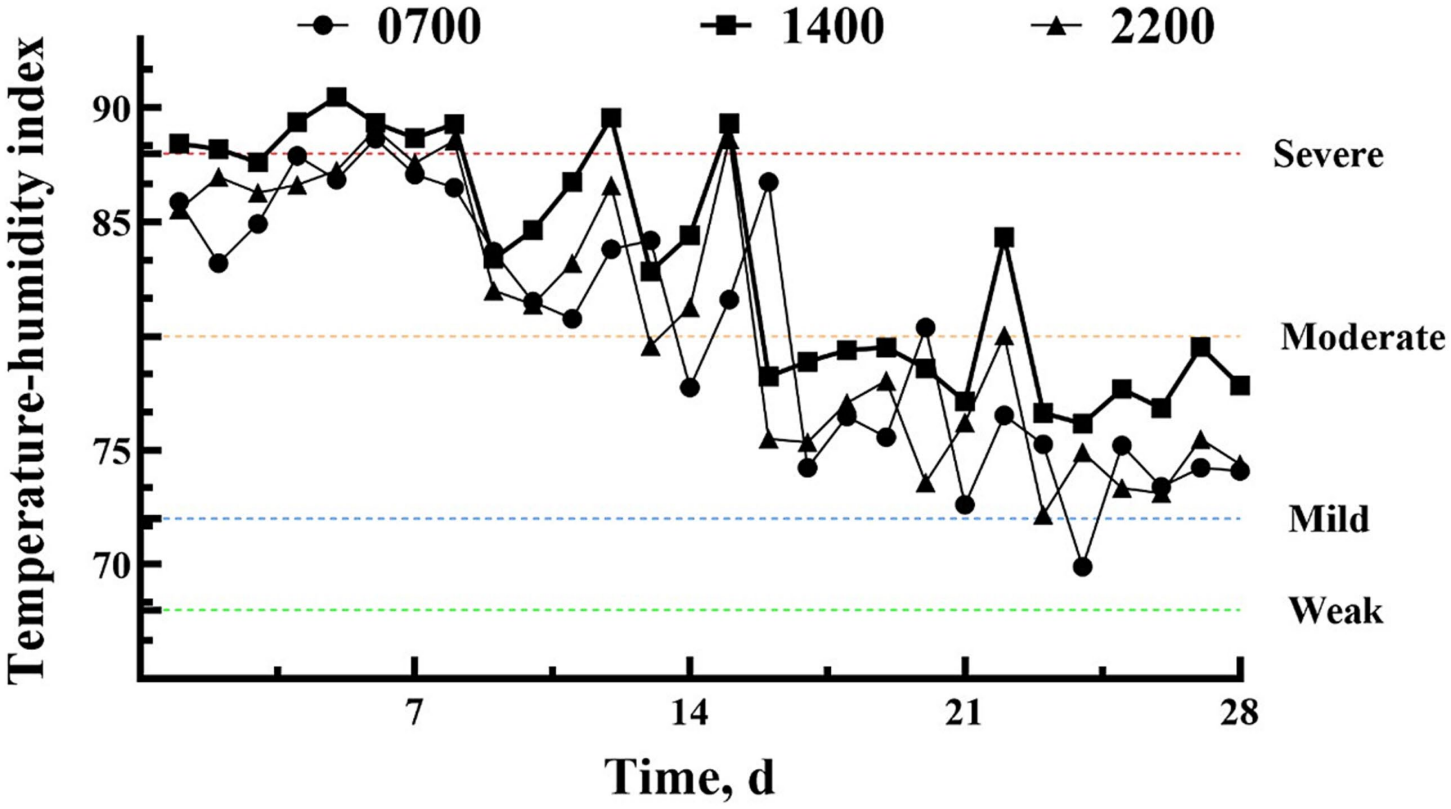
The temperature and humidity index in the cowshed at 0700 h, 1,400 h, and 1900 h during the experiment.

In the first 2 weeks of the experiment, dairy cows experienced severe (TH I > 88) to moderate heat stress (THI > 80) (Figure 1), gradually transitioning to moderate to mild heat stress (THI > 72) in the third and fourth weeks.

### 3.2 Effects on feed intake and milk yield

**Figure 2:**
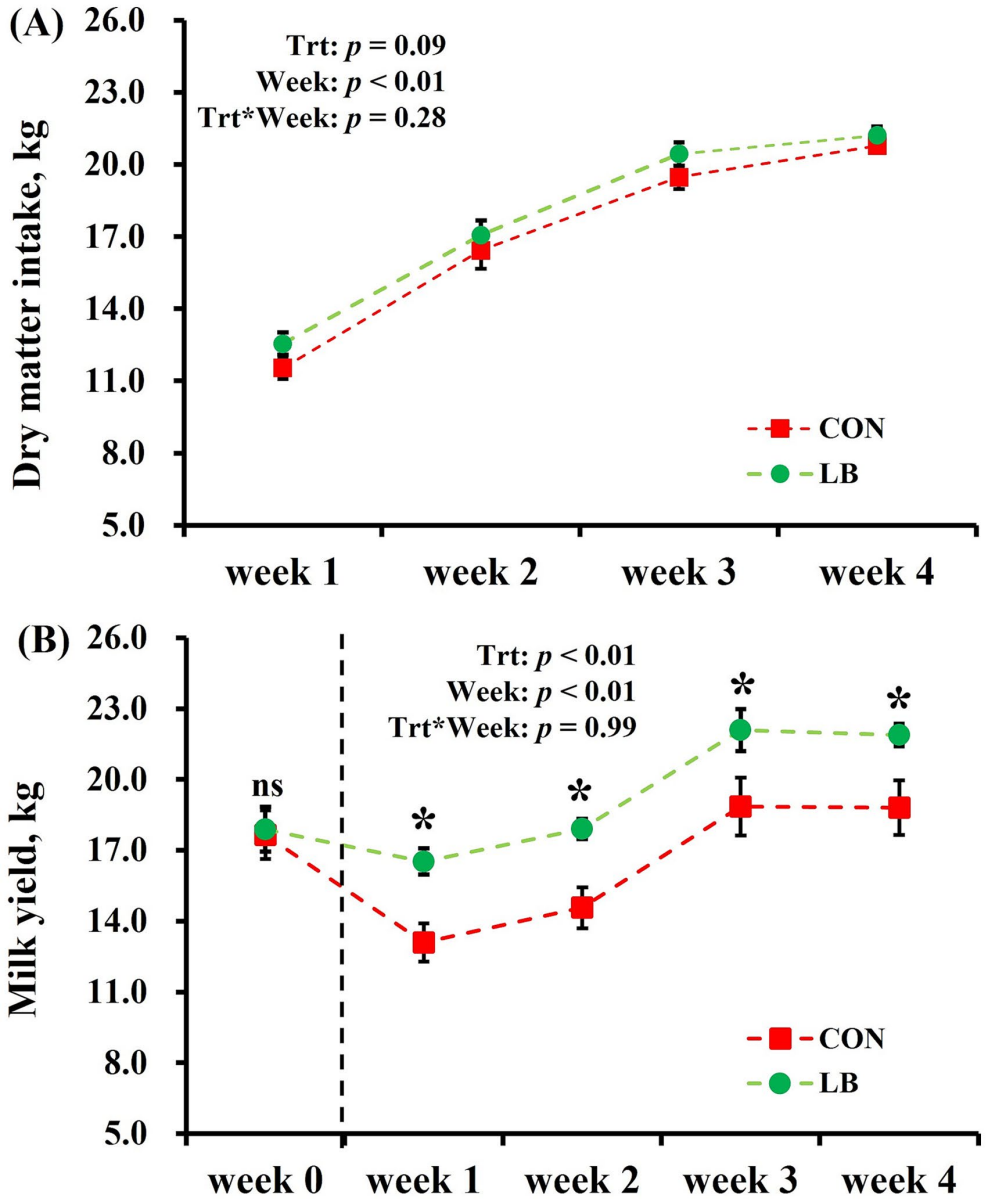
Dry matter intake **(A)** and Milk yield **(B)** of dairy cows offered and not offered access to HSCN lick block. *Indicates a statistically significant difference (*p* < 0.05).

There was no interaction between the weeks and treatment on the effects of DMI and milk yield (Figure 2). Both DMI and milk yield show an increase (week: *p <* 0.01) as the degree of heat stress decreased from the first to the fourth week (Figures 1, 2). There was no difference in the initial milk yield difference between two groups. Offer the HSCN lick block to dairy cows increased (*p <* 0.01) the milk yield from the first week to the fourth week (Figure 2A), with an increased tendency (*p* = 0.09) of DMI (Figure 2B).

### 3.3 Effects on milk composition of dairy cows

No interaction (*p* > 0.10) of week × treatment was detected on any of the analyzed milk fat, milk protein, milk lactose, total solids, and milk urea nitrogen of dairy cows (Table 2). Supplementation lick block increased (*p <* 0.05) the milk yield of dairy cows including 3.5% FCM and ECM without (*p* > 0.05) affecting milk composition, which led to an increase (*p <* 0.01) in the total synthesis of milk fat, milk protein, lactose, and total solids.

**Table 2:** Milk yield and composition of dairy cows offered and not offered access to HSCN lick block.

Item	Treatment		SEM	*p*- value		
	CON	LB		Trt	Week	Trt × Week
Milk yield, kg	16.33	19.60	0.496	<0.01	<0.01	0.99
3.5% FCM^1^, kg	16.48	19.11	0.418	<0.01	<0.01	0.89
ECM^2^, kg	16.71	19.44	0.398	<0.01	<0.01	0.94
Milk fat, %	3.47	3.31	0.092	0.23	<0.01	0.24
Milk protein, %	3.18	3.17	0.073	0.95	<0.01	0.78
Milk lactose, %	4.82	4.91	0.049	0.21	0.41	0.12
Milk solids, %	12.40	12.28	0.163	0.61	0.02	0.82
Milk fat yield, kg/d	0.627	0.753	0.019	<0.01	<0.01	0.99
Milk protein yield, kg/d	0.517	0.620	0.015	<0.01	<0.01	0.94
Milk lactose yield, kg/d	0.790	0.962	0.026	<0.01	<0.01	0.85
Milk solids yield, kg/d	2.019	2.406	0.054	<0.01	<0.01	0.98
Milk urea nitrogen, mg/dL	13.08	13.09	0.241	0.96	<0.01	0.07

^1^3.5% FCM = (0.432 + 0.165 × percentage of fat) × kg of milk (40). ^2^ECM = 0.327 × milk yield + 12.86 × fat yield + 7.65 × protein yield (41).

### 3.4 Effects on somatic cell counts and somatic cell scores

**Figure 3:**
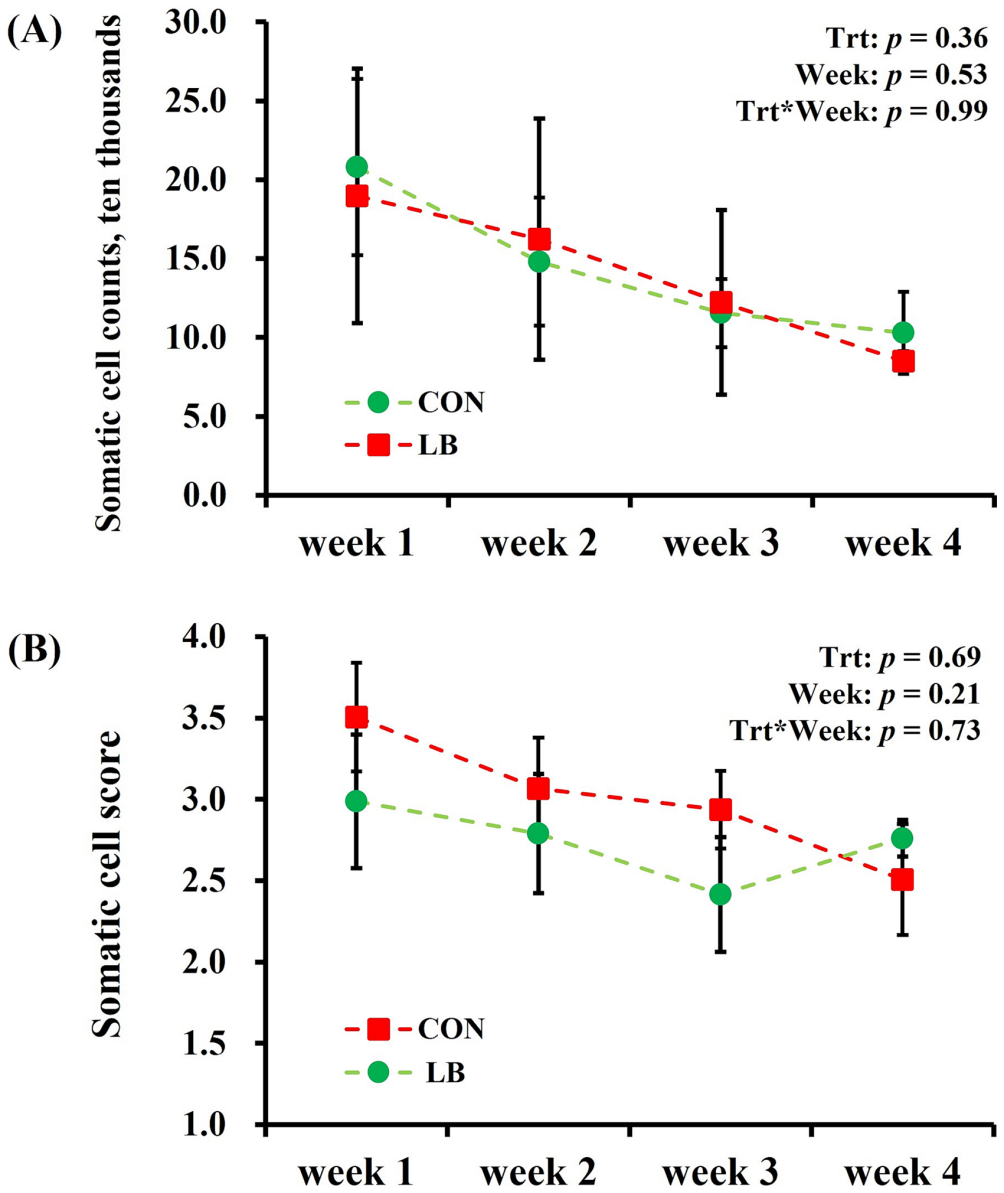
Somatic cell counts **(A)** and Somatic cell score **(B)** of dairy cows offered and not offered access to HSCN lick block.

No interaction (*p* > 0.10) of week × treatment was detected on any of the analyzed SCC and SCS of dairy cows (Figure 3). The SCC and SCS of milk did not differ between the first to fourth weeks of the experimental period.

**Table 3:** The rumen fermentation characteristics in dairy cows offered and not offered access to HSCN lick block.

Item	Treatment		SEM	*p*- value
	CON	LB		
pH	5.82	6.11	0.091	0.04
Ammonia, mg/dL	12.15	8.83	0.744	<0.01
Total VFAs, mM	99.37	92.94	2.200	0.07
Acetate, mM	59.34	58.58	1.330	0.69
Propionate, mM	21.52	18.32	0.931	0.035
A: P	2.80	3.21	0.142	0.07
Butyrate, mM	14.07	11.99	0.780	0.09
Valerate, mM	1.33	1.85	0.170	0.07
Isobutyrate, mM	0.753	0.760	0.060	0.94
Isovalerate, mM	1.35	1.44	0.229	0.79

### 3.5 Effects on respiratory rate and rectal temperature

**Figure 4:**
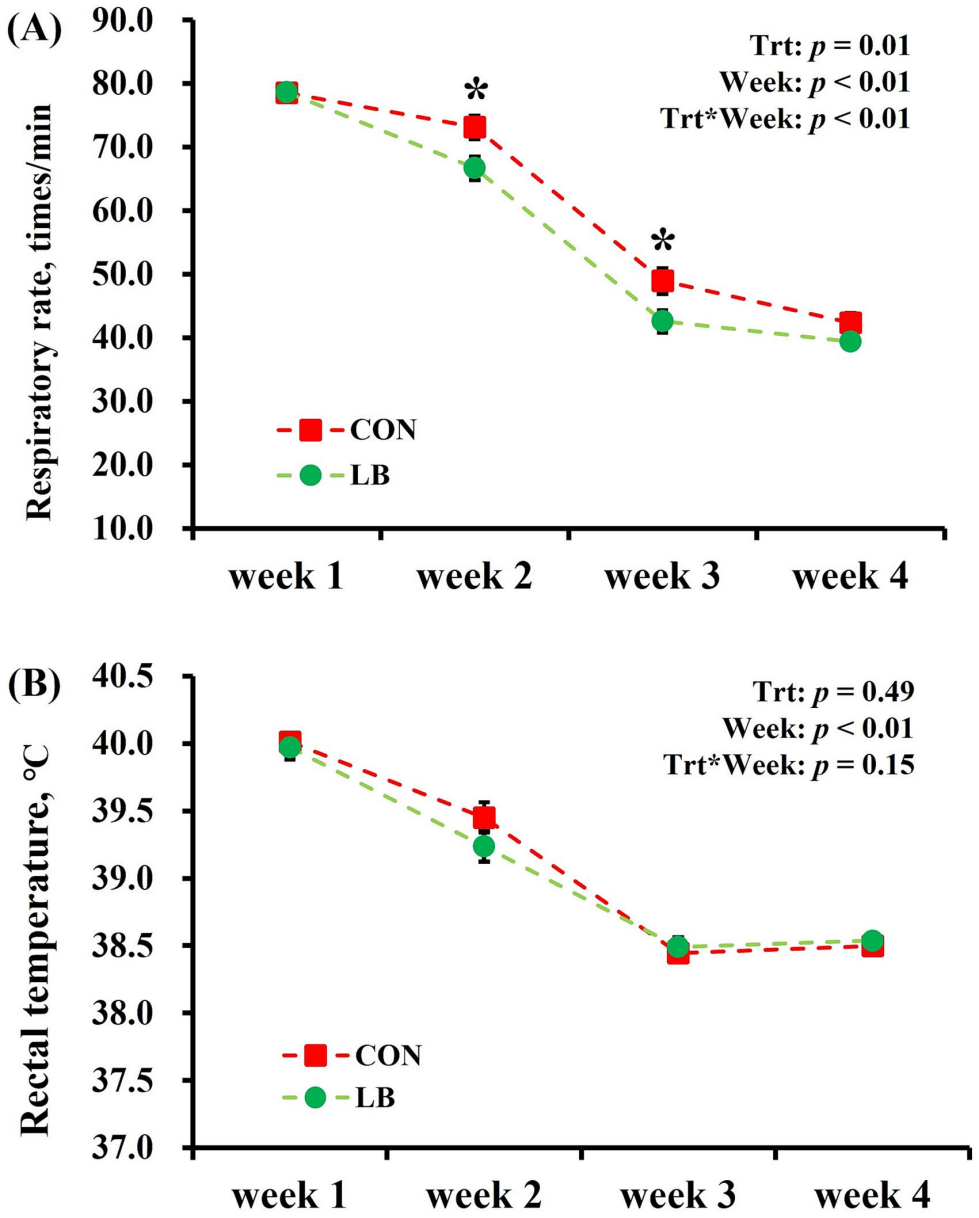
The respiratory rate **(A)** and rectal temperature **(B)** of dairy cows offered and not offered access to HSCN lick block. *Indicates a statistically significant difference (*p* < 0.05).

No interaction (*p* > 0.10) of week × treatment was detected on the RR, but there was an interaction (*p <* 0.05) of treatment × time on the RT (Figure 4). Independent analyses showed that the lick block group had lower (*p <* 0.05) RR than the control group in the second and third weeks. RT decreased (Week: *p <* 0.05) with the decrease in the degree of heat stress from the first to the fourth week, and no difference (*p* > 0.05) was observed in the RT between the two groups.

### 3.6 Effect on rumen fermentation parameters

Dairy cows offered access to an HSCN lick block had a greater rumen pH and lower NH_3_-N and propionate concentration (*p <* 0.05). Meanwhile, the acetate-to-propionate ratio (*p* = 0.07), total VFA concentration (*p* = 0.07), and butyrate concentration (*p* = 0.09) had a declining trend in the LB group (Table 3).

## 4 Discussion

Heat stress causes a sharp drop in feed intake, resulting in poor nutrient intake, decreased productivity, and poor offspring growth. It can also trigger inflammatory responses in the body that affect the health of dairy cows (1). When the THI exceeds 68, cows exhibit signs of heat stress such as elevated body temperature, increased respiratory rate, and water intake, while decreasing feed intake, and milk yield (27). This study incorporates specific ratios of potassium, selenium, chromium, zinc, vitamin A, and vitamin E, which have the potential to reduce heat stress, to formulate an anti-heat stress lick block. These blocks were provided for free access to cows potentially experiencing heat stress, with the aim of assessing the effectiveness of the anti-heat stress intervention.

During the duration of the experiment, the THI remained consistently between 69 to 90, signifying continuous heat stress exposure for dairy cows. Previous studies have shown that cows exposed to heat stress have significantly lower feed intake and milk yield. The higher the level of heat stress, the greater the decline in these metrics (28, 29). In the current study, dairy cows exposed to heat stress in the LB group had a higher feed intake and milk yield compared to the CON group, likely due to the lick block offered. This suggests that the heat stress experienced by the cows was mitigated. Additionally, a previous study indicated that heat stress negatively affects milk quality by lowering both milk fat and milk protein levels (30). In contrast, this study found that offering lick blocks to dairy cows enhanced the synthesis of milk fat and protein. This improvement was most likely related to the lower level of heat stress experienced by the cows. With a lower degree of heat stress, cows were more efficient in synthesizing milk fat and milk protein; meanwhile, the higher feed intake provided more substrates for the synthesis of milk fat and protein.

Somatic cell count serves as a crucial indicator for assessing the health status of dairy cows. Transforming somatic cell numbers to obtain a somatic cell score, which adheres more closely to a normal distribution and facilitates statistical analysis, is a common practice (31). A previous study demonstrated a significant negative correlation between somatic cell score and milk yield, indicating that higher somatic cell scores are associated with decreased milk production (32). In the current study, a lower somatic cell score corresponded to an increase in milk yield as heat stress lessened, corroborating the findings of Bellagi et al. (33). It is noteworthy that the LB group had a lower somatic cell score compared to the CON group for the first 3 weeks. Although this difference was only numerical, it suggested to some extent that heat stress in cows was alleviated.

A previous study revealed a notable increase in cows’ respiratory rate at high THI levels (34). Concurrently, the challenge of effectively dissipating the cows’ excess metabolic heat in such conditions leads to elevated body temperatures among dairy cows (35). Throughout the experimental duration, the cows experienced persistently high THI levels. Remarkably, lactating dairy cows offered access to HSCN block had a lower respiratory rate, potentially indicating that the provision of the lick block mitigated heat stress in the cows. However, contrary to expectations, there was no substantial variation in body temperature between two groups. This unexpected outcome may be attributed to the high barn temperatures during the experiment, which impeded effective heat dissipation.

Rumen homeostasis is crucial for ensuring the efficient digestion of nutrients and the overall health of ruminants. Rumen pH as an important indicator of rumen homeostasis influenced by multiple factors such as diet type, animal feeding and drinking, and animal health. Previously, a study highlighted the impact of heat stress on altering animals’ feed intake behavior, showing a decrease in forage intake and an increase in concentrate intake (36). Excessive concentrate intake can trigger a short-term surge in VFA production in the rumen, causing a rapid decline in rumen pH and escalating the risk of rumen acidosis. Additionally, the increased respiratory rate and decreased salivary secretion of dairy cows caused by heat stress will also decrease the concentration of carbonates available for exchange in blood and saliva, ultimately resulting in lower rumen pH (2). Supplementary lick blocks have previously been found to increase salivary secretion, which contains many buffering substances that help maintain rumen homeostasis (20). Meanwhile, the decline in respiratory rate allowed for more carbonates to be used for exchange in the blood entering the rumen, which may explain the elevated rumen pH.

The ratio of concentrate to roughage in the diet affects the content of acetate, propionate, and butyrate produced during rumen fermentation. When feeding diets with higher fiber levels, the total VFA in the rumen will decrease, while the pH and acetate levels will increase. In contrast, when the ratio of concentrate (starch) is increased, the proportion of propionate increases dramatically (37, 38). In the present study, although the proportion of concentrate and roughage consumed by the cows was not measured, dairy cows in LB group had a lower concentrations of rumen TVFA, propionate, and butyrate and a great concentration of acetate. This potentially indicates that dairy cows in the LB group exhibited less picky eating behavior and consumed more roughage, which was similarly reported in a study by Nonaka et al. (39). The supplementation of lick block alleviated the heat stress and improved the body and rumen health of the dairy cows, and the tendency to decrease the total acid concentration also may be related to the enhancement of rumen absorption function. Additionally, in the present study, the reduced ammonia-nitrogen concentration in the lick block treatment may be related to the improved rumen fermentation, which resulted in more ammonia being used to synthesize microbial proteins.

## 5 Conclusion

Supplementation with anti-heat stress lick block improved rumen fermentation and reduced the respiratory rate of dairy cows, resulting in increased feed intake, milk yield, and both milk fat and milk protein yields. Therefore, the supplementation of anti-heat stress lick block has a beneficial effect on alleviating heat stress in dairy cows and enhancing productivity.

## Data Availability

The raw data supporting the conclusions of this article will be made available by the authors, without undue reservation.
