# Supplementary material for: Anti-heat stress lick block supplementation alleviated the detrimental effects of heat stress on dairy cows
Source: Front Vet Sci. 2025 Mar 7;12:1562964. doi: 10.3389/fvets.2025.1562964 (PMC11927217; doi:10.3389/fvets.2025.1562964)
Supplement: Supplementary file 1 [file Table_1.docx]

Supplementary Material

# Supplementary Table S1: The composition of the anti-heat stress compound nutrition lick block

| Ingredient | Additive amount (kg/t) |
| --- | --- |
| Ca (H_2_PO_4_) _2_ | 1.27 |
| CaCO_3_ | 1.28 |
| MgO | 8.64 |
| KCl | 28.68 |
| CoCl_2_·H_2_O (1% net content) | 19.23 |
| Cu_2_(OH)_3_Cl | 0.86 |
| Ca (IO_3_)_2_ | 12.14 |
| FeSO_4_·H_2_O | 0.33 |
| Fe_2_O_3_ | 3.00 |
| MnSO_4_·H_2_O | 6.15 |
| Na_2_SeO_3_ (1% net content) | 5.59 |
| ZnO | 5.24 |
| C_9_H_15_CrO_6_ (1.4% Cr net content) | 1.07 |
| Ethoxyquin | 0.59 |
| Vitamin A | 0.40 |
| Vitamin D | 0.16 |
| Vitamin E (50%) | 4.00 |
| Vitamin C (96%) | 3.13 |
| Water | 4.50 |
| NaCl | 893.74 |
